# Establishment of reference values based on influential characteristics of hematopoietic stem cells and immune cell subsets in the bone marrow

**DOI:** 10.1016/j.heliyon.2024.e30888

**Published:** 2024-05-08

**Authors:** Rebar N. Mohammed, Najmaddin S.H. Khoshnaw, Vian Faeq Mohammed, Dastan O. Hassan, Chra Nawfal Abdullah, Tavan Ismael Mahmood, Huda A. Abbass, Dereen Ahmed, Kani D. Noori, Lanja I. Saeed, Salah Mohammed Salih, Hiwa S. Sidiq, Dlnya Omer Ali, Alan Shwan, Ignazio Majolino, Francesco Ipsevich

**Affiliations:** a Department of Medical Laboratory Technology, Faculty of Health Science, Qaiwan International University, Sulaimani, Kurdistan Region, Iraq; b College of Veterinary Medicine, University of Sulaimani, Sulaymaniyah, Iraq; c Bone Marrow Transplant Center, Hiwa Hospital, Sulaymaniyah, KRG, Iraq; d Department of Medical Laboratory Science, College of Science, Komar University of Science and Technology, KRG, Iraq; e Department of Clinical Science, College of Medicine, University of Sulaimani, Sulaymaniyah, Iraq; f Ospedale San Camillo and Salvator Mundi International Hospital, Rome, Italy

**Keywords:** Bone marrow graft, HSC, Immune cells, Healthy donor, Reference range

## Abstract

Hematopoietic stem cell transplantation is still a curative treatment for many haematological cancers. Many factors, such as age, sex, ethnic background, smoking status, and body mass index, affect average reference values in different populations. This study aimed to establish a reference range for the absolute numbers and percentages of healthy individuals' hematopoietic stem cells and immune cells in the bone marrow. Seventy-one healthy donors (32 males and 39 females) were enrolled in the study. Following bone marrow harvesting, using flow cytometry, immunophenotyping was performed to determine the absolute number and percentage of CD34^+^ stem cells and various immune subsets. We found no statistically significant difference in the absolute count of HSCs or immune cell subsets in the bone marrow between males and females. Regarding age, the younger group had more significant CD34^+^ and immune cell subsets. Donors with healthier body weights tend to have richer bone marrow cellularity. Establishing a reference value for hematopoietic stem cells and immune cells in the bone marrow based on various influential factors is pivotal for defining bone marrow status and donor selection.

## 1 Introduction

Hematopoietic stem cells (HSCT) are rare subsets of specialized hematopoietic cells responsible for the lifelong maintenance of bone marrow precursors and progenitor cells. HSCT remains the primary curative treatment for many haematological cancers and benign disorders, such as congenital marrow failure syndrome and thalassemia [1,2]. A successful HSCT with a better clinical outcome depends on the donor site stem cell count, based on the number of CD34^+^ cells or total nucleated bone marrow cells [3].

Among the various factors, age-related changes in the major lymphocyte subsets in bone marrow blood occur from childhood to adulthood [4]. Age-related changes were established as the main factor that helps to assess bone marrow lymphoid cell subsets [5].

Another factor that is important in considering donor selection is sex. In many medical aspects, such as immunocompetence and mortality, sex differences have been well documented in humans [6]. Many studies have shown that sex variations in donors and recipients affect HSCT outcomes [7]. For example, a study showed that HSCT in male recipients resulted in better transplant outcomes in terms of overall survival and disease-free progression survival than did HSCT in female recipients, regardless of donor sex [8,9].

Body mass index (BMI) has gained increased interest in recent decades since obesity has spread worldwide [10]. At the cellular level, an increase or decrease in BMI considerably influences homeostasis [11]. Many research studies have shown that BMI is directly related to blood cells, especially total white blood cells, particularly CD4^+^ and CD8^+^ lymphocytes [12]. Because obesity is more common in women, they tend to be most affected [12]. Therefore, determining the relationship between BMI and the cellular content of the bone marrow is of particular interest.

Immunophenotyping of stem cells and lymphocytes is essential in evaluating the immune system in health and disease [13]. Flow cytometry monitors blood components in patients with leukaemia, immunodeficiency syndrome, or lymphoma and evaluates immune status [14,15]. It is a reliable and accurate tool for rapidly and quantitatively determining cellular components [16]. Many factors, such as environmental factors, infection status, nutritional status, smoking status, sex, age, race, ethical diversity and immunosenescence, can affect the immune system. Therefore, establishing an internal normal range of HSCs and lymphocyte subsets is necessary for each population and worldwide [17,18]. Although similar methods were used to estimate lymphocyte subsets, there was still variation in the percentage of lymphocytes in different populations, as typical reference values may apply to one population but not others [19]. The current study aimed to determine reference values for HSCs and lymphocyte subsets in the bone marrow of healthy donors based on various influential factors, such as age, sex and BMI.

## 2 Materials and methods

### 2.1 HSCT center

This study was conducted at an HSCT centre (established June 2016) of Hiwa Hospital, Suleimanyah/Iraq, with six single-bed, HEPA-filtered, positive-pressure sterile rooms; four double-bed clean rooms; a bone marrow harvest surgical room; an apheresis unit; a manipulation laboratory for cell separation and cryopreservation; and a flow cytometry laboratory [20].

### 2.2 Bone marrow harvesting

After signing the informed consent and approval from the local ethics committee of the College of Medicine, University of Sulaimani, Sulaymaniyah, Iraq, with approval no. 7/29/701 meeting no. 4), bone marrow (BM) was harvested from healthy donors (n = 71) (Table 1). The BM graft was harvested from the posterior superior iliac crest. First, the harvesting point and surrounding areas were appropriately disinfected with 10 % iodine. Then, a sterile 13G trocar was introduced into the pelvic bone. After that, the bone marrow was harvested using a large syringe prefilled with heparinized normal saline (25,000 IU heparin in 500 ml NS). The graft was then transferred into a sterile bag and filtered in preparation for infusion into the patient. A 2 ml sample was prepared from the filtered BM graft and sent to the flow cytometry laboratory for HSC and immune cell evaluation and enumeration.

**Table 1:** Donor demographic characteristics.

Gender (n)	Male	32
	Female	39
Age Median (Range)	Child	7 (2–12)
	Adolescence	15 (13–18)
	Adult	29 (19–41)
BMI (kg/m^2^) Median (Range)	Underweight	
	Healthy	
	Overweight	
	Obese	

### 2.3 Cell enumeration by flow cytometry

HSC enumeration and viability were assessed using a stem cell enumeration kit (Cat # 664231, Lot # 3234923, BD Biosciences, Brea, CA) and FACS Via flow cytometer (BD Biosciences, USA). The procedure was performed with an accurate count (BD Biosciences, USA) containing known numbers of beads in a freeze-dried palette. For CD34 cell enumeration, 20 μl of stem cell reagent containing anti-CD45 FITC and anti-CD34 PE monoclonal antibodies and 20 μl of 7AAD reagent were added, and for immune cell enumeration, a cocktail of anti-CD3 FITC, anti-CD4 PE, and anti-CD45 Percp Cy5.5 and anti-CD8 APC (Cat # 342417, LOT # 09225 BD Biosciences, Brea, CA), or anti-CD3 FITC, anti-CD16/56 PE, anti-CD45 Percp Cy5.5 and anti-CD19-APC (Cat # 342416, LOT # 21623 BD Biosciences, Brea, CA), were added. Then, 100 μl of freshly harvested bone marrow graft was added by reverse pipetting. The sample was incubated for 20 min at room temperature in the dark. After that, 2 ml of 1x fixative free ammonium chloride RBC lysis buffer was added, and the sample was incubated for an additional 10 min at room temperature in the dark until all the RBCs were lysed. The samples were subsequently analyzed on a FACS VIA flow cytometer, and the data were analyzed with BD Accuri research software. The absolute numbers of CD34^+^ cells (HSCs), CD3^+^ T cells, CD3^+^CD4^+^ T cells, CD3^+^CD8^+^ T cells, CD19^+^ B cells, CD3^−^CD16/56^+^ NK cells and CD3^+^CD16/56^+^ NKT cells were calculated according to the manufacturer's protocol.

### 2.4 Flow cytometry quality control

The United Kingdom National External Quality Assurance System (UK NEQAS) accredited and continuously monitored the flow cytometry laboratory for CD34^+^ stem cell enumeration, CD34^+^ stem cell viability assessment and immune cell monitoring.

### 2.5 Reference interval verification

To establish the reference value, the Skewness-Kurtness test was first used to assess the normal distribution of the data. Then, the Box-Cox transformation was used to transform the non-normally distributed data. The healthy donors were grouped based on their age into Children (0–11 years old), Adolescents (12–17 years old) and adults (18 above years old) according to WHO age group classification. Furthermore, for the establishment of reference value based on gender, the donors were grouped into male and female and for the BMI, the donors were grouped into Underweight (below 18.5 kg/m^2^), Healthy (18.5–25 kg/m^2^), Overweight (25–30 kg/m^2^) and Obese (30-above kg/m^2^).

### 2.6 Statistical analysis

The Skewness-Kurtness test was used to assess the data's distribution and determine whether the data was usually or non-normally distributed. The Box-Cox transformation was used to transform the non-normally distributed data. Further analysis of the data was conducted in GraphPad Prism version 9.3.0. The reference values were obtained by identifying minimum and maximum values with the mean ± SEM (standard error of the mean) and the median with standard deviation (SD) of the absolute numbers and percentages of the cells using frequency distribution analysis.

## 3 Results

Reference values of sex-specific HSCs and immune cell subsets in the bone marrow of healthy donors.

To establish the reference values of sex-related HSCs and immune cell subsets in the bone marrow, 71 healthy donors (male, n = 32; female, n = 39) who donated their marrow grafts to their recipient patients were included in the study. The absolute numbers and percentages of CD34^+^ HSCs were in line with the absolute counts and percentages of lymphocyte subpopulations, including CD3^+^ T cells (total and further assessment of CD3^+^CD4^+^ and CD3^+^CD8^+^ T cells), CD19^+^ B cells, CD3^−^CD16/56^+^ NK cells and CD3^+^CD16/56^+^ NKT cells. The reference values were obtained by identifying minimum and maximum values with the mean±SEM (standard error of the mean) and the median with standard deviation (SD) of the absolute numbers and percentages of the cells of the harvested marrow. Moreover, we explored the variation and influence of sex on the number of bone marrow cells. Based on our investigation, there were no statistically significant differences in the absolute counts of HSCs or immune cell subsets in the bone marrow of male and female individuals, which suggests that sex is not a pivotal influencer of the number of progenitor and immune cells in the bone marrow (Table 2).

**Table 2:** Hematopoietic stem cell and immune cell subset normal values and characteristics based on sex in the bone marrow of healthy donors (absolute numbers and percentages).

Parameters	group	Mean	SEM	Median	SD	Normal Values
HSC (abs)	All	216.9	10.9	211.3	93.0	71–552
	Male	220.2	16.3	221.9	90.7	81–430
	Female	214.5	14.8	202.3	95.7	71–552
HSC (%)	All	0.30	0.01	0.30	0.08	0.10–0.50
	Male	0.29	0.01	0.30	0.09	0.16–0.50
	Female	0.30	0.01	0.30	0.08	0.10–0.50
Lymphocyte (cells/μl)	All	6130	381	5272	3169	1356–17109
	Male	5822	493	5219	2789	1356–13533
	Female	6175	527	5170	3418	1968–17109
Lymphocyte (%)	All	9.68	0.42	9.00	3.42	5.00–18.00
	Male	9.21	0.50	9.00	2.63	6.00–15.00
	Female	10.00	0.62	9.00	3.87	5.00–18.00
T cells (cells/μl)	All	3794	189	3486	1630	1006–8814
	Male	3793	287	3465	1626	1006–8814
	Female	3795	255	3610	1653	1279–7817
T cells (%)	All	64.63	1.13	65.5	9.22	39.00–85.00
	Male	65.74	1.24	66.00	6.45	53.00–78.00
	Female	63.87	1.72	64.00	10.75	39.00–85.00
CD4^+^ T cells (cells/μl)	All	1773	89	1613	767	460–3768
	Male	1702	123	1563	696	460–3442
	Female	1827	126	1696	822	610–3768
CD4^+^ T cells (%)	All	47.10	0.97	46.5	7.90	34.00–65.00
	Male	46.18	1.63	45.00	8.47	34.00–63.00
	Female	47.74	1.20	48.00	7.53	34.00–65.00
CD8^+^ T cells (cells/μl)	All	1553	89	1494	736	422–4154
	Male	1545	119	1494	676	423–2927
	Female	1516	122	1320	793	518–4154
CD8^+^ T cells (%)	All	40.40	0.87	40.00	7.13	25.00–56.00
	Male	40.48	1.37	39.00	7.14	28.00–56.00
	Female	40.35	1.15	40.00	7.22	25.00–55.00
B cells (cells/μl)	All	1308	113	1114	926	259–4808
	Male	1181	132	1063	751	259–2817
	Female	1319	159	1006	1021	298–4804
B cells (%)	All	20.87	0.87	20.00	7.12	5.00–44.00
	Male	19.77	1.09	20.00	5.70	10.00–31.00
	Female	21.64	1.27	21.00	7.94	5.00–44.00
NK cells (cells/μl)	All	765	69	527	557	89–2619
	Male	782	96	526	546	89–2286
	Female	725	84	552	549	114–2619
NK cells (%)	All	12.21	0.51	12.00	4.21	3.00–22.00
	Male	12.96	0.85	13.00	4.44	3.00–22.00
	Female	11.69	0.64	11.00	4.02	3.00–22.00
NKT cells (cells/μl)	All	311	19	265	162	52–730
	Male	326	29	285	163	52–624
	Female	299	25	252	162	78–730
NKT cells (%)	All	5.40	0.33	5.00	2.74	1.00–12.00
	Male	5.92	0.49	6.00	2.58	1.00–11.00
	Female	5.05	0.45	5.00	2.82	1.00–12.00

### 3.1 Establishment of reference values for HSCs and lymphocytes from the bone marrow according to age

Furthermore, to identify the reference values of HSCs and lymphocyte subpopulations in the bone marrow of healthy individuals based on age, 38 children, 15 adolescents and 18 adults were recruited for the study. The absolute numbers and percentages of CD34^+^ cells, CD3^+^ T cells, CD3^+^CD4^+^ cells, CD3^+^CD8^+^ T cells, CD19^+^ B cells, CD3^−^CD16/56^+^ NK cells and CD3^+^CD16/56^+^ NKT cells were calculated. The reference values are the minimum and maximum ± SEM and the median ± SD. Several parameters differed according to the age group. Compared with adults, children had significantly greater numbers of CD34^+^ and CD3^+^ T cells; CD3^+^CD4^+^ and CD3^+^CD8^+^ T cells; and CD19^+^ B cells and CD3^−^CD16/56+ NK cells. The differences were statistically significant compared to those in the adult group, except for the CD3^+^CD16/56+ NKT cells. However, compared with adolescents, children were significantly different only in the number of CD34^+^ cells, total CD3^+^ T cells, total CD19^+^ B cells, and total CD3^−^CD16/56+ NK cells. HSCs and lymphocyte subpopulations in the bone marrow tended to be higher in the adolescent group than in the adult group (Table 3).

**Table 3:** Hematopoietic stem cell and immune cell subset normal values and characteristics based on age in the bone marrow of healthy donors (absolute numbers and percentages).

Parameters	Groups	Mean	SEM	Median	SD	Normal Values
HSC (cells/μl)	Child	255.9	13.9	245.1	85.7	117.9–552.4
	Adolescence	176.7	12.2	173.4	48.9	97.8–254.4
	Adult	175.9	24.9	143.9	105.8	71.4–430.2
HSC (%)	Child	0.32	0.01	0.30	0.08	0.18–0.50
	Adolescence	0.26	0.01	0.24	0.07	0.16–0.40
	Adult	0.30	0.02	0.28	0.08	0.18–0.43
Lymphocyte (cells/μl)	Child	7779	520	7195	3206	2659–17109
	Adolescence	5016	413	4331	1653	3529–9072
	Adult	3560	331	3112	1443	1356–6165
Lymphocyte (%)	Child	11.02	0.57	11.00	3.54	5.00–18.00
	Adolescence	7.88	0.53	7.00	2.01	5.00–11.00
	Adult	8.04	0.75	8.00	2.51	6.00–15.00
T cells (cells/μl)	Child	4568	251	4767	1552	1764–8814
	Adolescence	3494	350	2941	1400	1406–6557
	Adult	2612	243	2462	1061	1006–4616
T cells (%)	Child	60.21	1.09	60.00	6.75	39.00–72.00
	Adolescence	70.42	2.02	71.50	7.57	54.00–85.00
	Adult	73.09	1.60	75.00	5.33	64.99–83.00
CD4^+^ T cells (cells/μl)	Child	2049	115	2016	713	605–3557
	Adolescence	1603	171	1415	663	610–2980
	Adult	1302	126	1333	552	460–2712
CD4^+^ T cells (%)	Child	44.81	1.28	43.00	7.94	34.00–65.00
	Adolescence	49.50	2.14	51.00	8.00	34.00–63.00
	Adult	52.27	1.66	52.00	5.53	43.00–59.00
CD8^+^ T cells (cells/μl)	Child	1842	125	1763	776	604–4154
	Adolescence	1370	148	1167	592	566–2808
	Adult	1078	105	1002	457	422–1832
CD8^+^ T cells (%)	Child	41.07	1.22	40.00	7.52	25.00–55.00
	Adolescence	39.71	2.30	40.00	8.61	30.00–56.00
	Adult	38.45	1.21	37.00	4.03	34.00–45.00
B cells (cells/μl)	Child	1789	156	1583	950	345–4808
	Adolescence	968	102	976	410	313–1634
	Adult	518	66	430	102	259–1319
B cells (%)	Child	23.73	1.00	24.00	6.18	12.00–44.00
	Adolescence	17.92	1.76	17.00	6.59	5.00–30.00
	Adult	14.72	1.09	15.00	3.63	9.00–22.00
NK cells (cells/μl)	Child	1049	93	962	579	211–2619
	Adolescence	505	74	445	296	121–1383
	Adult	391	48	363	210	89–973
NK cells (%)	Child	13.23	0.70	13.00	4.33	3.00–22.00
	Adolescence	9.92	0.87	10.00	3.29	3.00–15.00
	Adult	11.90	1.19	12.00	3.96	8.00–20.00
NKT cells (cells/μl)	Child	337	27	333	169	52–730
	Adolescence	169	32	266	130	82–522
	Adult	27	38	218	169	99–618
NKT cells (%)	Child	4.68	0.42	4.00	2.62	1.00–12.00
	Adolescence	5.85	0.61	6.50	2.31	2.00–9.00
	Adult	7.54	0.73	7.00	2.42	5.00–12.00

### 3.2 The influence of BMI on the cellularity of bone marrow in terms of HSCs and immune cell subpopulations

**The** BMI has attracted increased interest in medical research in recent decades. Therefore, the BMI of the healthy donors was calculated as the weight in kilograms (kg) divided by the square of the height in meters (m^2^), and the donors were subsequently categorized into underweight, healthy, overweight and obese based on their BMI. The reference values are the minimum and maximum ± SEM and the median and SD of HSCs and bone marrow lymphocyte numbers and percentages, respectively. We found that the body mass indices of all three age groups affect the number of bone marrow stem cells and immune cell subsets in the bone marrow of healthy donors. Our investigation shows donors with healthier body weights and even underweight individuals have more significant absolute numbers of HSCs and mature immune cells in the bone marrow than overweight and obese donors (Table 4).

**Table 4:** Hematopoietic stem cell and immune cell subset normal values and characteristics based on BMI in the bone marrow of healthy donors (absolute numbers and percentages).

Parameters	Groups	Mean	SEM	Median	SD	Normal Values
HSC (cells/μl)	Underweight	251	14.92	241	88.29	113–552
	Healthy	191	17.81	180	75.56	81–370
	Overweight	187	27.75	182	87.76	76–321
	Obese	117	22.11	87	49.45	71–177
HSC (%)	Underweight	0.34	0.02	0.32	0.06	0.28–0.47
	Healthy	0.28	0.01	0.26	0.08	0.16–0.50
	Overweight	0.27	0.02	0.27	0.09	0.16–0.43
	Obese	0.32	0.02	0.32	0.08	0.20–0.45
Lymphocyte (cells/μl)	Underweight	7837	535.7	7367	3169.3	2659–17109
	Healthy	4815	518.6	4022	2260.5	2562–11294
	Overweight	4604	582.4	4558	1931.7	2230–9072
	Obese	3154	834.7	2099	1866.5	1356–5272
Lymphocyte (%)	Underweight	8.68	0.67	8.75	1.90	5.00–11.00
	Healthy	9.24	0.54	9.00	2.73	5.00–15.00
	Overweight	8.07	0.51	8.00	1.84	6.00–12.00
	Obese	7.87	0.66	7.50	1.88	6.00–10.00
T cells (cells/μl)	Underweight	4706	255.1	4835	1509.4	1764–8814
	Healthy	3135	283.8	2742	1237.3	1788–6253
	Overweight	3244	392.8	3317	1302.8	1681–6077
	Obese	2256	627.1	1552	1402.1	1006–4248
T cells (%)	Underweight	60.25	1.88	59.00	5.33	53.00–68.00
	Healthy	66.72	1.63	69.00	8.18	53.00–85.00
	Overweight	67.23	2.46	67.00	8.87	52.00–83.00
	Obese	68.50	2.23	68.00	6.32	60.00–80.00
CD4^+^ T cells (cells/μl)	Underweight	2117	131.4	2024	777.4	605–3768
	Healthy	1572	133.8	1436	583.6	831–2849
	Overweight	1560	201.3	1464	667.9	769–2792
	Obese	1111	296.2	792	662.3	460–1857
CD4^+^ T cells (%)	Underweight	43.37	1.81	43.50	5.12	35.00–50.00
	Healthy	47.64	1.61	48.00	8.07	35.00–63.00
	Overweight	49.23	2.05	50.00	7.40	34.00–59.00
	Obese	47.25	1.67	46.50	4.74	40.00–56.00
CD8^+^ T cells (cells/μl)	Underweight	1908	123.1	1908	728.5	797–4154
	Healthy	1201	125.3	1059	546.1	604–2757
	Overweight	1335	184.1	1396	610.3	727–2808
	Obese	944	294.7	628	659.1	422–2015
CD8^+^ T cells (%)	Underweight	41.12	2.12	39.50	6.01	33.00–52.00
	Healthy	40.32	1.35	40.00	6.77	30.00–55.00
	Overweight	39.84	1.72	38.00	6.20	33.00–56.00
	Obese	41.12	1.79	42.00	5.08	32.00–47.00
B cells (cells/μl)	Underweight	1747	173.3	1595	1010.4	313–4804
	Healthy	985	136.5	797	595.3	313–2766
	Overweight	781	138.1	557	458.2	259–1518
	Obese	591	165.2	358	369.4	277–1012
B cells (%)	Underweight	23.25	1.27	23.50	3.61	18.00–29.00
	Healthy	19.04	1.21	20.00	6.07	5.00–29.00
	Overweight	18.00	1.62	18.00	5.84	9.00–28.00
	Obese	18.62	1.79	17.00	5.06	14.00–30.00
NK cells (cells/μl)	Underweight	1009	90.7	876	536.9	211–2619
	Healthy	619	122.4	418	533.8	238–2153
	Overweight	545	106.8	475	354.4	121–1383
	Obese	252	111.1	152	248.2	89–688
NK cells (%)	Underweight	15.00	1.70	15.00	4.81	8.00–22.00
	Healthy	12.52	0.73	12.00	3.68	6.00–22.00
	Overweight	13.46	1.32	13.00	4.78	8.00–20.00
	Obese	10.00	1.30	10.00	3.70	3.00–15.00
NKT cells (cells/μl)	Underweight	354	26.2	362	153.1	52–730
	Healthy	252	31.3	220	136.5	78–624
	Overweight	354	55.9	322	185.4	119–618
	Obese	114	8.4	106	18.8	99–145
NKT cells (%)	Underweight	5.12	0.76	5.50	2.16	2.00–8.00
	Healthy	5.80	0.49	5.00	2.46	2.00–12.00
	Overweight	6.61	0.85	6.00	3.09	2.00–12.00
	Obese	6.25	0.81	6.50	2.31	2.00–9.00

## 4 Discussion

Lymphocyte immunophenotyping is crucial for assessing the immune system because ethnic variety and environmental influences impact the immune system, and each population must have an internal normal range for its lymphocyte subsets [21]. Numerous cell subpopulations' concentrations and proportions vary according to age, sex, geography, and ethnicity. Understanding the typical levels of these cells and how they differ in healthy populations will help advance clinical practice for more accurate diagnosis and prognosis and scientific study [22]. Numerous studies have examined racial variations in the distributions of distinct lymphocyte subpopulations [23].

Data obtained from different nations not only demonstrate heterogeneity in the number of lymphocytes and their subsets but also, despite the use of comparable methodologies, reference ranges from studies conducted in one population do not apply to others [24].

Our study is the only study in the region in which lymphocyte subsets were evaluated based on BM samples, and the majority of the other studies are based on PB lymphocyte subsets; moreover, no distinct studies on BM-based samples could be detected; for this reason, we compare our results with comparable results in PB samples from other countries.

In our study, there was no noticeable difference in lymphocyte count or subset of lymphocytes between males and females, apart from mild deviation in the total lymphocyte count, absolute CD4^+^ T cell and CD19^+^ B cell count and percentage count, which were slightly greater in females than in males but were not significant. Based on age, all the lymphocyte subsets showed a dramatic reduction in count with increasing age, being highest in the childhood period and then in Adolescence and lowest in adulthood, which agrees with the findings of studies performed in Saudi Arabia in all age groups [25]. The T cell population (CD3^+^) was threefold more significant than the B cell population (CD19^+^) in all age groups, both males and females. Still, this ratio increased with age until it reached fivefold greater in adulthood. This result also matches that in Saudi Arabia and Iran [21,25] but does not match those obtained from previous studies in which the B cells were equivalent to the T cells in average adult BM [26]. The CD4:CD8 ratio is approximately 1:1 in males and females and all age groups; this ratio is slightly lower than that reported in previous studies [21,27,28] but by that of Saudi Arabia [25]. NK cells (CD56^+^) were nearly in the same range in all age groups and in both males and females, which is compatible with the results of other studies [21,27,28].

Compared to our neighbouring country (Iran), our result is more significant for all the subsets, especially for CD8^+^ T cells and CD19^+^ B cells, in both adult males and females [21]. However, compared to the results for Saudi Arabia, our results are more significant regarding the absolute counts of all the subsets. At the same time, they are nearly consistent in terms of their percentages. The percentage and absolute count of NK cells (CD56^+^) were lower in our adult group than in their study [25]. Additionally, our lymphocyte subset range is greater than that of several European (Germany), Asian (Hong Kong, Korea), Latin American (Peru, Cuba, Brazil) and African (Tanzania) countries, apart from CD19^+^ cells, which are the same as those of adult Cuban individuals [27]. These findings support that notional lymphocyte subset values are significantly influenced by age, sex, race, and environmental factors [28]. Since more significant donor-site stem cell counts are generally believed to lead to better clinical outcomes, these stem cell counts are essential [29].

There is constant trafficking of hematopoietic stem cells between the PB and extravascular marrow spaces. Consequently, it is not believed that the stem cell qualities of the BM and PB stem cell pools differ [30]. The variation in the cellularity component of the bone marrow with age relies on the ontological changes that occur in the bone marrow niche composition [31]. For instance, the developmental stages from foetal life to neonatal to adult life led to changes in extracellular matrix markers, proliferation capability and other developmental potentials, affecting the differences in average values [32,33]. The cellularity, proliferation capacity, trophic factor generation and differentiation potential of HSCs and lymphocytes in the bone marrow have yet to be well studied. One study showed that age, but not sex, impacts bone marrow cellularity; males and females have similar proportions of HSCs and mature lymphocytes in the bone marrow [34].

Many reports disagree about the influence of age on the CD34^+^ cell count, and some studies have reported that the CD34^+^ cell count decreases with increasing age [35]. Other works have shown that the functionality of HSCs decreases with increasing age, but the number of CD34^+^ cells is not strongly affected by increasing age [36]. Several others have reported that the CD34^+^ population in the bone marrow is expanding in individuals over 70 years [35]. Several reports have shown that the fraction of CD34^+^ cells in the bone marrow decreases during childhood, possibly due to the increased percentage of lymphocytes in the BM during age [37]. This variation in CD34^+^ cell quantification performed in different centres makes comparing different reports difficult [37]. It has been reported in some studies that the CD34^+^ cell count is inversely associated with female sex and positively associated with body mass index [38].

BMI has a significant role in determining the CD34^+^ cell count and mobilization, and the majority of reports indicate that the greater the BMI and obesity are, the more critical the CD34^+^ cell count [35]. BMI and obesity are associated with various adverse health issues, ranging from cardiovascular disease to diabetes type II, dyslipidemia, arthritis, hypertension, asthma and overall poor health conditions [39]. Our study's results showed no significant difference in the percentage or absolute count of CD34^+^ cells between males and females. Still, the absolute count was more important in children than adults, which agrees with the results of previous studies [29]. However, in our study, the percentage of CD34^+^ cells was the same in all age groups, but the absolute count was more significant in children than adults. These findings prove that BM cellularity is more important in the pediatric age group than in the adult age group [40]. Our results for CD34^+^ cell counts are lower than those recorded in previous studies, in which the median percentage of CD34^+^ cells was 1.1 %, while that in our study was 0.3 % [29,37].

Additionally, the median percentage of CD34^+^ cells in other studies was significantly more significant in children and adolescents (1.38 %) than in adults (0.62 %). In contrast, in our study, the median percentage in all age groups was 0.3 % [29]. Our study showed no difference in the range of CD34^+^ cell counts between the sexes, and these results are compatible with those of other studies [37].

## 5 Conclusion

Establishing a reference value for hematopoietic stem cells and immune cells in the bone marrow based on various influential factors is pivotal for donor selection and defining the bone marrow status.

## Availability of data

The datasets used and/or analyzed during the current study are available from the corresponding author upon reasonable request.

## Informed consent

The donors agreed on the bone marrow harvest procedure by signing an informed consent form.

## Ethical approval

The procedure performed in this study was approved by the College of Medicine Research Committee, University of Sulaimani, Kurdistan Regional Government, Kurdistan, Iraq (approval no. 7/29/701 meeting no. 4).

## Statement of human and animal rights

All procedures performed in this study involving human participants were ethically approved by the College of Medicine Research Committee, University of Sulaimani, Kurdistan Regional Government, Kurdistan, Iraq.

## CRediT authorship contribution statement

**Rebar N. Mohammed:** Writing – review & editing, Writing – original draft, Visualization, Validation, Supervision, Software, Methodology, Formal analysis, Data curation, Conceptualization. **Najmaddin S.H. Khoshnaw:** Writing – review & editing, Writing – original draft, Formal analysis, Conceptualization. **Vian Faeq Mohammed:** Writing – review & editing, Visualization, Validation, Supervision, Data curation, Conceptualization. **Dastan O. Hassan:** Writing – review & editing, Validation, Formal analysis, Data curation. **Chra Nawfal Abdullah:** Writing – review & editing, Writing – original draft, Validation, Formal analysis, Data curation. **Tavan Ismael Mahmood:** Writing – review & editing, Project administration, Investigation, Formal analysis, Data curation. **Huda A. Abbass:** Writing – review & editing, Writing – original draft, Software, Conceptualization. **Dereen Ahmed:** Writing – review & editing, Methodology, Formal analysis, Data curation. **Kani D. Noori:** Writing – review & editing, Writing – original draft, Visualization, Investigation, Conceptualization. **Lanja I. Saeed:** Methodology, Formal analysis, Conceptualization. **Salah Mohammed Salih:** Writing – review & editing, Validation, Methodology, Data curation. **Hiwa S. Sidiq:** Methodology, Formal analysis, Data curation. **Dlnya Omer Ali:** Visualization, Formal analysis, Data curation. **Alan Shwan:** Writing – review & editing, Writing – original draft, Software, Resources, Data curation, Conceptualization. **Ignazio Majolino:** Writing – review & editing, Visualization, Validation, Supervision, Formal analysis, Data curation, Conceptualization. **Francesco Ipsevich:** Writing – review & editing, Validation, Supervision, Investigation, Formal analysis, Data curation, Conceptualization.

## Declaration of competing interest

The authors declare that they have no known competing financial interests or personal relationships that could have appeared to influence the work reported in this paper.
